# Conceptualization of Surrogate Decision-making Among Spokespersons for Chronically Ill Patients

**DOI:** 10.1001/jamanetworkopen.2022.45608

**Published:** 2022-12-08

**Authors:** Lauren J. Van Scoy, Michael J. Green, Theresa Smith, Erika VanDyke, Andrew J. Foy, Laurie Badzek, Benjamin H. Levi

**Affiliations:** 1 Department of Medicine, Penn State College of Medicine, Hershey, Pennsylvania; 2 Department of Humanities, Penn State College of Medicine, Hershey, Pennsylvania; 3 Department of Public Health Sciences, Penn State College of Medicine, Hershey, Pennsylvania; 4 Ross and Carol Nese College of Nursing, Pennsylvania State University, University Park; 5 Department of Pediatrics, Penn State College of Medicine, Hershey, Pennsylvania

## Abstract

**Importance:**

The value of advance care planning (ACP) has been the subject of recent debate because of mixed findings. This may be, in part, because trials presume that researchers and patient spokespersons share the same understanding of the role of a surrogate decision-maker. We explored how patient surrogates conceptualized and defined surrogate decision-making vs patient advocacy. Understanding how surrogates perceive their role in decision-making is important to avoid misinterpreting the effectiveness of ACP interventions.

**Objective:**

To understand how patient spokespersons distinguish surrogate decision-making from patient advocacy.

**Design, Setting, and Participants:**

This qualitative thematic analysis of a subsample of participants from a randomized clinical trial at a tertiary medical center was conducted from September 27, 2012, to June 30, 2021. Participants (n = 36) were the designated spokespersons of adult patients with severe illness who had made a surrogate decision on behalf of the patient since the last follow-up. Analysis was performed from March 21, 2021, to February 7, 2022.

**Main Outcomes and Measures:**

Semistructured interviews examined how patient spokespersons conceptualize differences between surrogate decision-making and advocacy.

**Results:**

The study included 36 patient spokespersons (32 women [88.9%]; mean [SD] age, 62.1 [11.8] years) and found substantial variability in how the spokespersons conceptualized what it means to make a surrogate decision for another. A total of 10 spokespersons (27.8%) did not distinguish surrogate decision-making from advocacy. There were 5 definitions for both surrogate decision-making and advocacy. The 3 most common definitions of surrogate decision-making were (1) acting as the final decision-maker (18 [50.0%]), (2) doing what is best for the patient (8 [22.2%]), and (3) making decisions on behalf of patients so that their wishes are respected (6 [16.7%]). The 3 most common definitions of advocacy were (1) doing what is best for the patient (8 [22.2%]), (2) respecting patients’ wishes (6 [16.7%]), and (3) providing support to the patient (6 [16.7%]). The most common pairing of definitions by an individual spokesperson involved defining surrogate decision-making as being the final decision-maker, and defining advocacy as acting in the best interest of the patient (6 [16.7%]).

**Conclusions and Relevance:**

This qualitative study found that many spokespersons perceive their roles as surrogate decision-makers differently than clinicians and researchers likely do, often conflating surrogacy with advocacy. These findings may help explain why researchers have found that ACP does not consistently improve traditional outcomes. If spokespersons do not distinguish surrogate decision-making from advocacy, then what is being reported by spokespersons and measured by clinicians and researchers may not accurately reflect the true association of ACP with outcomes.

## Key Points

**Question:**

How do patient spokespersons distinguish surrogate medical decision-making from patient advocacy?

**Findings:**

This qualitative study of a subset of 36 participants in a randomized clinical trial found substantial variability in how spokespersons for patients with chronic illness conceptualize their role as surrogate decision-makers and/or patient advocates.

**Meaning:**

Understanding how spokespersons perceive their role is critical for assessing the effectiveness of advance care planning interventions.

## Teaser

This qualitative study examines how patient spokespersons distinguish surrogate decision-making from patient advocacy.

## Introduction

Advance care planning (ACP) is a process that includes considering one’s preferences for medical treatments, choosing a surrogate decision-maker, discussing preferences for medical care with that spokesperson as well as one’s health care professionals, and documenting them in an advance directive.^1^ Advance care planning is intended to help improve the process of surrogate decision-making for patients and their spokespersons, reduce burdens on the health care system, and improve the likelihood that patients receive goal-concordant care.^2^ Randomized clinical trials of ACP interventions have shown success for some of these outcomes, but not all.^2,3,4^ Systematic reviews covering decades of research have found heterogeneity in interventional approaches, trial outcomes, and participant samples, which likely contribute to these mixed findings.^2,3,5,6^ Disputes about the effectiveness of ACP at achieving goal-concordant care has led some to reject ACP altogether in favor of in-the-moment decision-making,^7^ and although “lack of evidence” does not equate to “lack of value,” the complexity of accurately gauging the effectiveness of ACP presents significant scientific challenges.^8^

In the course of studying spokespersons’ reported surrogate decision-making during a large, randomized clinical trial, a substantial discrepancy was found between the way surrogates conceptualized surrogate decision-making and the way clinicians and researchers conceptualized it. Furthermore, we discovered during early qualitative interviews that spokespersons may have conceptual differences in how they perceive their role, considering it to overlap with the concept of advocacy vs making surrogate decisions. This difference is important because ACP trials and outcome measures presume that researchers, clinicians, patients, and spokespersons share the same understanding of what is considered a surrogate decision. Even within our own multidisciplinary research team, we uncovered disagreements about what constitutes a surrogate decision. This “measurement” problem may help explain some of the discrepant findings from the ACP literature.

As such, we generated new research questions: how do spokespersons conceptualize surrogate decision-making, and is that conceptualization distinct from patient advocacy? To better understand spokespersons’ interpretations of what it means to make a surrogate decision, we added qualitative interview questions to the trial’s final study visit. What follows is a qualitative analysis of our findings.

## Methods

### Study Design

**Figure 1.**
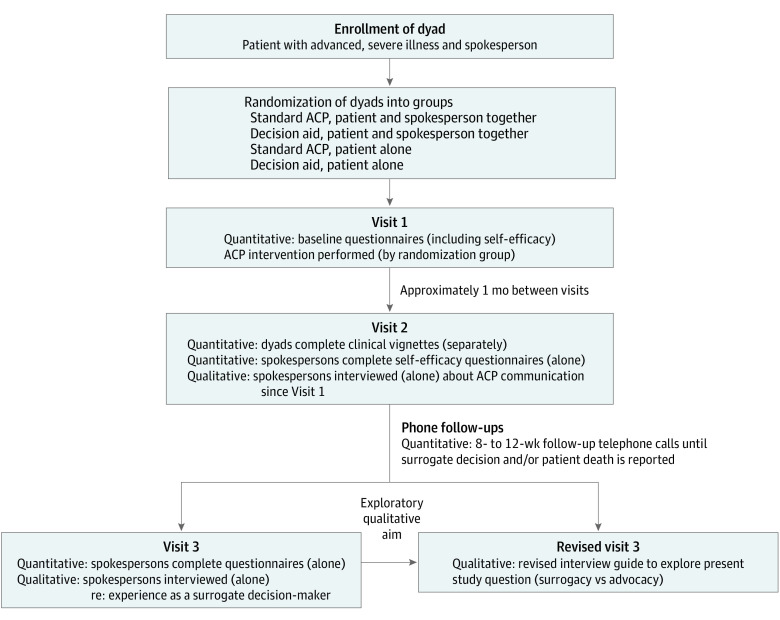
Study Design of the Parent Trial and Mixed-Methods Analysis ACP indicates advance care planning.

This qualitative study, conducted from September 27, 2012, to June 30, 2021, presents the second phase of an explanatory sequential mixed-methods analysis^9^ that arose from findings obtained during a large, 5-year, multiphase, randomized clinical trial examining the efficacy of an ACP decision aid (NCT02429479). The primary objective of the parent study was to evaluate whether the intervention better prepared spokespersons to make surrogate decisions for seriously ill patients. Physicians referred potential participants from outpatient subspecialty clinics using disease-specific inclusion criteria related to late-stage cancer, congestive heart failure (CHF), chronic obstructive pulmonary disease (COPD) or pulmonary fibrosis (PF), or end-stage kidney disease (ESKD) (for detailed inclusion criteria, see eMethods 1 in Supplement 1). Patients who provided written informed consent were asked to identify a study partner who was their spokesperson (defined as the person responsible for making their medical decisions if they were to become incapacitated). All participants completed questionnaires reporting basic demographic characteristics and other measures as part of the parent trial. Dyads were recruited from a tertiary care center with a wide rural catchment area (Hershey, Pennsylvania), and 2 urban hospitals (Harrisburg, Pennsylvania, and Boston, Massachusetts). Figure 1 illustrates the 3-visit trial. Throughout the trial, the coordinator contacted spokespersons (who also provided written informed consent) periodically to determine if they had recently made a surrogate decision on behalf of the patient. The study was approved by the Penn State College of Medicine institutional review board and adheres to reporting by the Consolidated Criteria for Reporting Qualitative Research (COREQ) reporting guideline (eMethods 2 in Supplement 1).^10^

**Figure 2.**
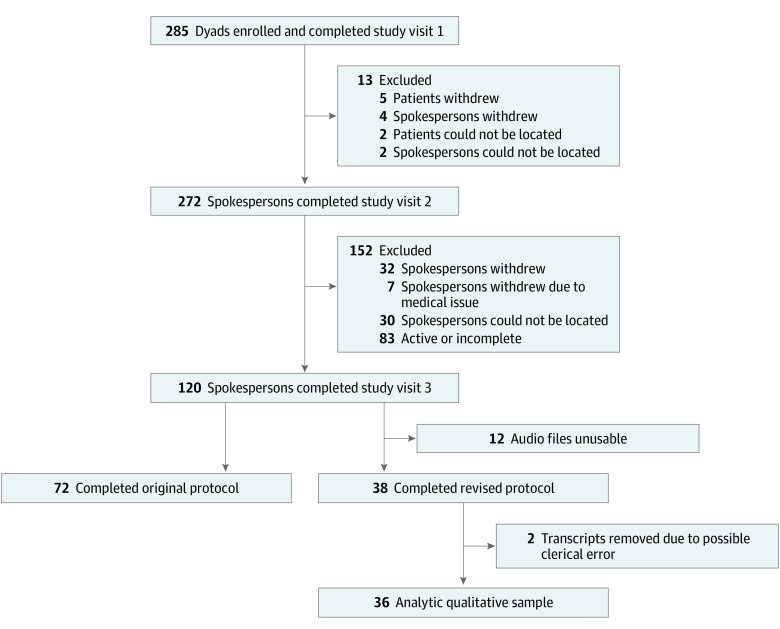
Diagram of Analytic Sample

To examine the variability in how participants interpreted “surrogate decision,” we adapted the final in-person qualitative interview to include questions exploring spokespersons’ understanding of surrogate decision-making. Figure 2 shows the parent study Consolidated Standards of Reporting Trials (CONSORT) diagram and how the qualitative sample (n = 36) was derived. The time elapsed from visit 2 to visit 3 (the interview for this study) was variable based on participant preferences, grieving, and availability and ranged from 3 weeks to 13 months. Interviews were conducted in private offices at the study site, participants’ homes (if preferred), or by videoconference (during the COVID-19 pandemic).

### Working Definition of a Surrogate Medical Decision

The initial study design defined “surrogate decision-maker” as a substitute health care decision-maker who consents to or refuses to consent to some or all medical treatments for the patient who lacks decision-making capacity.^11^ In telephone interviews every 3 to 4 months after the study intervention, patient spokespersons were asked if they had made a surrogate decision for the patient, in which case a final study interview was arranged.

At weekly research meetings when reported decisions were reviewed, questions soon arose whether particular decisions were in fact surrogate decisions or, for example, patient advocacy. As these questions became more frequent, we recognized the need to better characterize these emerging discrepancies, so we adapted the final interview to explore these nuances.

### Qualitative Interview and Analysis

The final study visit interview originally explored spokespersons’ perceived preparedness, stress, and (if applicable) bereavement experiences after having made a surrogate decision (eMethods 3 in Supplement 1). The following questions and prompts were then added: “People sometimes have very different understandings of what it means to make a surrogate decision. I’d like to learn what this concept means to you. In your own words, please tell me what ‘making a surrogate medical decision’ means to you.” Also, “Do you perceive that there is a difference between making a surrogate decision and being an advocate? If so, can you tell me more about that?”

Analysis was performed from March 21, 2021, to February 7, 2022. All interviews were audio-recorded, transcribed verbatim, and coded or analyzed by 2 experienced qualitative analysts (L.J.V.S. and E.V.) using MAXQDA 2020 (VERBI) software.^12^ We used a descriptive and inductive phenomenologic approach to content analysis.^13^ At the outset of coding, analysts discussed intrinsic biases related to surrogate decision-making and bracketed these to the extent possible to maintain neutrality. Additional qualitative rigor is described in eMethods 2 in Supplement 1.^14^

After reviewing 27.8% of transcripts (10 of 36), a preliminary codebook was created. Emerging concepts and codes (including definitions of “surrogate decision-making” and “advocacy”) were then identified. These codes were defined, and exemplar quotations were chosen to represent each code. Then, 2 analysts (L.J.V.S. and E.V.) applied this codebook (eTable in Supplement 1) to 25 transcripts using the constant comparison method.^15^ For instances in which individuals expressed more than 1 definition for a term, both codes were included. Cohen κ reports were used to adjudicate coding discrepancies (after calibration, κ was 0.98). Data saturation was achieved after reviewing 20% of transcripts; however, all transcripts were coded to allow for examination of patterns within subgroups. Then, analysts created themes and subthemes based on coding patterns. Associations between codes were assessed using the Code Relations Browser (MAXQDA) to quantify the intersection of occurrences of each definition (code) within a transcript. Frequency and percentages of coding intersections per individual were tabulated.

## Results

### Participant Characteristics

We analyzed 36 transcripts (32 female participants [88.9%] with a mean [SD] age if 62.1 [11.8] years); participant characteristics are shown in Table 1. Content analysis identified 5 definitions of surrogate decision-making and 5 definitions of advocacy (Table 2).

**Table 1.** Demographic Characteristics of Spokespersons

Characteristic	Spokespersons, No. (%) (N = 36)
Gender	
Female	32 (88.9)
Male	4 (11.1)
Age, mean (SD), y^a^	62.1 (11.8)
Race and ethnicity	
African American	9 (25.0)
Hispanic or Latino	0
White	27 (75.0)
Patient disease category	
Cardiac	11 (30.6)
Pulmonary	9 (25.0)
Cancer	8 (22.2)
Kidney	8 (22.2)
Education	
High school graduate or GED	9 (25.0)
Some college or technical school	17 (47.2)
College graduate	7 (19.4)
Graduate or professional school	3 (8.3)
Current marital status	
Never married	3 (8.3)
Married	29 (80.6)
Divorced or legally separated	3 (8.3)
Domestic partnership	1 (2.8)
Current employment status	
Employed	
Part time	4 (11.1)
Full time	14 (38.9)
Not currently employed and not seeking work	4 (11.1)
Not currently employed but seeking work	3 (8.3)
Retired	9 (25.0)
Other	2 (5.6)

Abbreviation: GED, General Educational Development certification.

^a^ Age at the time of visit 3.

**Table 2.** Spokespersons’ Definitions of Surrogate Decision-making and Advocacy

Definition	No. (%) (N = 36)^a^
Definitions of surrogate decision-making	
Being the final decision-maker	18 (50.0)
Doing what is best for patient	8 (22.2)
Fulfilling or respecting a patient’s wishes	6 (16.7)
Being the voice of the patient	4 (11.1)
Other	3 (8.3)
Scientific process	3 (8.3)
Definitions of advocacy	
Doing what is best for the patient	8 (22.2)
Respecting wishes of the patient	6 (16.7)
Providing support to the patient	6 (16.7)
Being the voice of the patient	4 (11.1)
Asking questions	4 (11.1)
Other	4 (11.1)

^a^ Participants may have endorsed more than 1 definition (double coding allowed).

### Five Ways Spokespersons Defined Surrogate Decision-making

#### Definition 1: Being the Final Decision-maker

The most frequent definition of surrogate decision-making was the spokesperson “making the final decision” on behalf of another person who cannot do so themselves:

To me [being a surrogate decision-maker] means that I have a decision to make, not only for myself but for my mother and also on behalf of my siblings (Child of patient with COPD).

[M]aking a surrogate medical decision is [when patient] is not capable of making that decision for himself. That’s where I step in (Spouse of patient with ESKD and CHF).

When explaining the “final decision,” most spokespersons referenced the patient’s mental status or capacity when defining a surrogate decision:

Making a decision on behalf of someone who can’t mentally or verbally make it on their own (Child of patient with ESKD).

At that point I was making the decision. This answer will be final, mine will be the answer, I don’t care what he says, you are not giving this to him (Spouse of patient with CHF).

“Final decisions” that participants described referred to either procedural interventions (eg, surgery) or end-of-life care:

I would have to make a decision as far as any procedures concerned, I would consider that a surrogate decision, as well as any type of update to his code status (Spouse of patient with CHF).

And, honestly, when he’s collapsed on the floor…not able to, to communicate. I mean, that’s when I have to make a decision. Pull the plug or don’t pull the plug (Spouse of patient with ESKD and CHF).

Many spokespersons also conceptualized surrogate decision-making as being the person who must make difficult choices and actually implement those final decisions:

I guess being the decision-maker is actually following through with it (Child of patient with end-stage cancer).

I really see surrogacy as sometimes…making hard decisions that you might not want (Child of patient with COPD).

#### Definition 2: Doing What Is Best for the Patient

The second most common definition of surrogate decision-making involved implementing what spokespersons felt was “best” for the patient, broadly framed, but with the implicit assumption that there was 1 best option:

For me to make a surrogate decision means to make the best decision for the person…whether it’s a mental health issue, a physical health, or financial decision. You make the best decision for that person with unconditional love and not for any benefit and it’s got to be a decision that you will not regret (Child of patient with ESKD).

What counted as the best decision sometimes was framed paternalistically, such that the goal of benefitting a patient was distinct from the patient’s own wishes:

[Surrogate decisions involve] things that are important…even though she doesn’t want [them]. We try and talk her into…making the decision that this is, this is what you need…whether you like it or not. This is what you need (Child of patient with COPD).

#### Definition 3: Respecting Patient’s Wishes

The third most common definition of surrogate decision-making involved respecting patient autonomy, which entailed knowing and respecting the patient’s wishes:

I think it’s definitely knowing…what they want…what their wishes are, in case…their partner or whomever wants to go against their wishes. So [the] surrogate is like, “No, this is, this is what we talked about. This is what he wants,” and making sure it stays that way (Child of patient with end-stage cancer).

It’s making a decision when they can’t make the decision, and making the decision of what they would want even if it wasn’t what you wanted (Spouse of patient with end-stage cancer).

#### Definition 4: Voice of Patient

In a slight contrast with the theme of “respecting patient’s wishes,” some spokespersons described surrogate decision-making as serving as the patient’s voice, with a particular emphasis placed on “speaking up” for a patient:

That means being [their] voice when they cannot speak (Friend of patient with ESKD).

When he gets ill, he gets quiet. When I get mad, I get quiet. But when I am sick, I am going to tell you why I am. He’s not like that, so I really feel I have to speak on his behalf (Spouse of patient with CHF).

#### Definition 5: A Scientific Process

Other spokespersons described surrogate decision-making as using a scientific process to consider the pros and cons of various medical options:

You find out all you can about the situation…and then look at benefits and disadvantages…and you decide, okay, this is the one that we’ll go with…based on the data you have at the time…Make it as scientific as possible…When you go down through the criteria and you get to the bottom, and your decision point says this is the decision, then that’s it. [Y]ou make it, and you don’t look back (Spouse of patient with CHF).

### Five Ways Spokespersons Defined Advocacy

When asked to define advocacy, many of the themes that emerged were similar to those of surrogate decision-making. However, a given spokesperson typically described the concepts differently—with different terms and a different emphasis on elements of the role being played.

#### Definition 1: Doing What Is Best for the Patient

Advocacy was most commonly described as fighting for what is in the patient’s best interest or doing the “right thing”:

Sometimes she knows she needs things, like with her hand…she [said] “Oh, it’ll heal. It’ll heal.” I’m like, “No…We’re going [to the doctor]” (Child of patient with COPD).

[If] I’m just giving advice [then I’m a surrogate, but if] I’m fighting for the right thing for you, then I’m…an advocate. I guess that’s how I would differentiate [them] (Child of patient with COPD or PF).

#### Definition 2: Fulfilling or Respecting Patient’s Wishes

When advocacy was framed in terms of fulfilling a patient’s wishes, it was often comingled with being their voice and doing what’s best for the patient. That said, this theme emerged less frequently than when surrogate decision-making was being described:

I would see as an advocate definitely being like, “This is what he wants” (Child of patient with COPD or PF).

Being the advocate is trying to do what they want, respect their wishes (Spouse of patient with COPD).

#### Definition 3: Providing Support

Some spokespersons described advocacy as more personal than surrogate decision-making, involving emotional and/or moral support for the patient’s decisions:

Being an advocate…[involves being] more supportive of what he wants to do, and being behind him for his thoughts and desires (Child of patient with COPD or PF).

I see advocating as supporting the person (Child of patient with COPD).

#### Definition 4: Being the Voice of the Patient

Some spokespersons described advocacy in terms of speaking up on behalf of the patient, particularly when the patient chooses not to do so:

When we go to the doctor, she gets upset with me sometimes because I tell her straight up. “I’m not going to lie for you! If they ask a question and you just say, No…If I know different, I am going to tell them…” [An] advocate [speaks] up and says, “Look, this is really what’s going on” (Child of patient with COPD).

Actually, you’re being their voice even if they have their voice (Child of patient with end-stage cancer).

#### Definition 5: Asking Questions

Similar to how some spokespersons defined surrogate decision-making in terms of gathering information, many spokespersons described advocacy in terms of asking questions. In doing so, however, advocacy was also framed in terms of both pushing for answers (or additional options) and providing health information to the clinical team:

I was always an advocate for him…I wanted him to get whatever he needed. I called the coordinator if there was a problem, and…there is always more questions when you get a heart pump (Spouse of patient with coronary heart disease).

You have to be an advocate to make the right decision. If you’re not pushing for all the answers…and you just kind of say, okay, that sounds good…you’re not getting all the information from that person (Spouse of patient with CHF).

I would say the small difference is that being an advocate, it’s still the other person’s decision…You can ask the questions that the patient may not think of to ask (Spouse of patient with CHF).

### Association Between Definitions of Surrogate Decision-making and Advocacy

To examine the way an individual spokesperson defined surrogate decision-making vs advocacy, definitions were assembled in a cross-tab matrix (Table 3) showing how a given spokesperson defined surrogate decision-making and how that same person defined advocacy.

**Table 3.** Matrix of Combinations for How a Given Spokesperson Defined Surrogate Decision-making and How That Same Person Defined Advocacy^a^

Surrogate decision-making definitions (column)	Advocacy definitions (row), No. (%) (N = 36)						Total No.	
	Best interest of patient	Voice of patient	Respecting wishes	Providing support	Asking questions	Other	Codes^b^	Individuals
Final decision-maker	6 (16.7)	3 (8.3)	4 (11.1)	3 (8.3)	2 (5.6)	3 (8.3)	21	18
Fulfilling or respecting patient’s wishes	2 (5.6)	1 (2.8)	3 (8.3)	1 (2.8)	0	1 (2.8)	8	6
Best interest of patient	3 (8.3)	2 (5.6)	1 (2.8)	2 (5.6)	1 (2.8)	0	9	8
Scientific process	1 (2.8)	1 (2.8)	0	2 (5.6)	1 (2.8)	0	5	3
Voice of patient	0	0	1 (2.8)	0	0	2 (5.6)	3	4^c^
Other	1 (2.8)	0	0	0	1 (2.8)	1 (2.8)	3	3
Total No.								
Codes	8	7	9	8	5	4	NA	NA
Individuals	8	4	6	6	4	4	NA	NA

Abbreviation: NA, not applicable.

^a^ Cells represent the number of times individual participants defined surrogate decision-making (rows) with a definition of advocacy (columns). For example, there were 6 individuals who defined surrogate decision-making as being a final decision-maker while also defining advocacy as doing what is in the best interest of the patient (16.7%).

^b^ Codes may be overlapping or more than 1 code per individual.

^c^ One individual did not have a codable advocacy definition.

The most common pairing of definitions involved defining surrogate decision-making as being the final decision-maker and definding advocacy as acting in the best interest of the patient (9 [16.7%]). For example, 1 participant said:

To me making a [surrogate] decision is…a question of [choosing] A or B. Being an advocate is saying, “Okay, neither A nor B is acceptable…What else can you do to make him better? He’s chosen no to both of these. So, what else is there?” (Spouse of patient with ESKD and CHF).

A total of 10 spokespersons (27.8%) did not distinguish between surrogate decision-making and advocacy:

I think it’s the same thing. Because it’s what’s best for him. It’s always…about him (Child of patient with CHF).

Several spokespersons reported difficulty articulating differences between surrogate decision-making and advocacy because they had to play both roles at the same time:

They definitely overlap. You play both roles at the same time. That’s the only way I can put it (Child of patient with CHF).

Well, I was both. Because when something wasn’t right I fought for it, I did something about it. And, otherwise I was there with him helping make decisions (Spouse of patient with COPD).

## Discussion

The present findings reveal substantial variability in how spokespersons for patients with advanced illness conceptualized what it means to make a surrogate decision, with significant overlap in how they understood surrogate decision-making and advocacy. A total of 10 spokespersons (27.8%) did not distinguish surrogate decision-making from advocacy. The 3 most common interpretations of what surrogate decision-making means included (1) acting as the final decision-maker (18 [50.0%]), (2) doing what is best for the patient (8 [22.2%]), and (3) making decisions on behalf of patients so that their wishes are respected (6 [16.7%]). The 3 most common interpretations of advocacy included (1) doing what is best for the patient (8 [22.2%]), (2) helping make sure patient’s wishes are respected (6 [16.7%]), and (3) providing support to the patient (6 [16.7%]). The most common pairing of interpretations for a given spokesperson was defining surrogate decision-making as acting as the final decision-maker for the patient and defining advocacy as doing what is in the best interest of the patient (6 [16.7%]).

These findings may help explain why our research team and others have found that ACP does not consistently improve either surrogate decision-makers’ self-efficacy or spokesperson-patient concordance regarding end-of-life treatment wishes.^4,16^ When there is conflation between surrogate decision-making and advocacy, the outcomes being measured and reported may likewise become jumbled. Because ACP interventions are typically designed to prepare spokespersons to make surrogate decisions, it should come as no surprise that such interventions do not help people feel prepared to advocate for patients. But if surrogate decision-making and advocacy are not distinct concepts in the minds of spokespersons, then what is being reported by spokespersons and measured by clinicians and researchers may not accurately reflect true surrogate decision-making. So, too, if the lived reality of spokespersons is that surrogate decision-making and advocacy are often inseparable, perhaps ACP interventions should also help prepare people for being an advocate. Either way, when assessing an intervention’s effectiveness at helping spokespersons make surrogate decisions that align with patient preferences, we must ensure that how spokespersons understand their role as surrogate decision-makers is aligned with the outcomes that we are measuring.

In our study, “facilitating goal-concordant care” was seen as the goal of surrogate decision-making by only 6 spokespersons (16.7%) compared with 8 spokespersons (22.2%) who defined surrogate decision-making in terms of “doing what’s best” for the patient. As such, our data suggest that ACP researchers should identify measures that reflect how surrogates understand the tasks of decision-making and advocacy. If that were accomplished, successful ACP might extend beyond facilitating goal-concordant care and also include helping potential decision-makers support and advocate for their loved ones.

From this analysis and other data collected during the trial, we learned that, in practice, many supposed surrogate decisions are actually instances of advocacy or an amalgam of advocacy and surrogate decision-making. Recognizing such conflation (and the need for more precise terminology and measurement strategies) is important for avoiding errors in assessing ACP interventions. Thus, the present findings can help inform the field of ACP research to avoid both type I and type II errors that risk undermining the true benefits of ACP.

### Limitations

This study has some limitations, including that the respondents were predominantly women. The sample of participants was from only 2 northeastern areas and was not racially and ethnically diverse, which may limit generalizability to other regions. Furthermore, there is opportunity for recall bias from participants because the interviews occurred at varied times after decision-making had occurred. Selection bias may also influence findings because individuals more interested in research or ACP are more likely to participate in this research.

## Conclusions

In light of renewed debate over the value of the ACP, how best to study and prepare patient spokespersons remains unclear. What is clear, however, is that to be an effective spokesperson for patients who cannot make their own medical decisions, most people need help preparing for that role, which often has multiple elements. To the extent that ACP can be expanded to help spokespersons with both surrogate decision-making and advocacy and the researchers can accurately measure its association with various outcomes, evidence-based ACP interventions can likely help support patients and their spokespersons when they are often at their most vulnerable.

## Notes

Supplement 1. **eMethods 1.** Inclusion/Exclusion Criteria From Parent Trial **eMethods 2.** Description of Adherence to the 32 Items of the Consolidated Criterion for Reporting Qualitative Research (COREQ) **eMethods 3.** Visit 3 Interview Guide Revised Versions **eTable.** Coding Tree

Supplement 2.
Data Sharing Statement
