# Supplementary material for: Conceptualization of Surrogate Decision-making Among Spokespersons for Chronically Ill Patients
Source: JAMA Netw Open. 2022 Dec 8;5(12):e2245608. doi: 10.1001/jamanetworkopen.2022.45608 (PMC9856522; doi:10.1001/jamanetworkopen.2022.45608)
Supplement: Supplement 1. — eMethods 1. Inclusion/Exclusion Criteria From Parent Trial eMethods 2. Description of Adherence to the 32 Items of the Consolidated Criterion for Reporting Qualitative Research (COREQ) eMethods 3. Visit 3 Interview Guide Revised Versions eTable. Coding Tree [file jamanetwopen-e2245608-s001.pdf]

Supplementary Online Content

Van Scoy LJ, Green MJ, Smith T, et al. Conceptualization of surrogate decision-making among spokespersons for chronically ill patients. *JAMA Netw Open.* 2022;5(12):e2245608. doi:10.1001/jamanetworkopen.2022.45608

eMethods 1. Inclusion/Exclusion Criteria From Parent Trial
eMethods 2. Description of Adherence to the 32 Items of the Consolidated Criterion for Reporting Qualitative Research (COREQ)
eMethods 3. Visit 3 Interview Guide Revised Versions
eTable. Coding Tree

This supplementary material has been provided by the authors to give readers additional information about their work.

© 2022 Van Scoy LJ et al. *JAMA Network Open.*

eMethods 1. Inclusion/Exclusion Criteria From Parent Trial

Inclusion Criteria

Patient inclusion criteria are:

1. >18 years of age
2. Diagnosis of kidney disease (e.g. chronic kidney disease, end stage renal disease) or advanced cancer (Stage IV disease or having an estimated survival of <2 years) or severe heart failure (e.g. New York Heart Assoc. Class III or Class IV) or severe lung disease (e.g. Stage III or Stage IV COPD by modified GOLD Spirometric Classification, Idiopathic Pulmonary Fibrosis) 75
3. Able to read and understand English at an 8th grade level (word 26 on either blue or tan version of the WRAT-3 reading subtest)

4) Neuro-cognitively able to engage in ACP (Mini Mental State Exam score >23)
5) No active suicidal ideations (i.e., score of 0 or 1 on item 9 of the BDI-II).

Caregiver inclusion criteria:

1) >18 years of age
2) Identified by the patient as a family caregiver who will be the patient's surrogate decision-maker (if non-cohabitant, must have in-person interaction with patient at least once weekly)
3) Able to read and understand English at an 8th grade level (word 26 on either blue or tan version of the WRAT-3 reading subtest)
4) Neuro-cognitively able to engage in ACP (Mini Mental State Exam score >23)

Exclusion Criteria

Patient exclusion criteria are:

1) <18 years of age
2) Unable to read and understand English at an 8th grade level (<26 on either blue or tan version of the WRAT-3 reading subtest)
3) Neuro-cognitively unable to engage in ACP (Mini Mental State Exam score ≤23)
4) Suicidal ideations or actively considering suicide (score of 2 or 3 on item 9 of the BDI-II)

Caregiver exclusion criteria:

1) <18 years of age
2) Unable to read and understand English at an 8th grade level (<26 on either blue or tan version of the WRAT-3 reading subtest)
3) Neuro-cognitively unable to engage in ACP (Mini Mental State Exam score ≤23)

© 2022 Van Scoy LJ et al. *JAMA Network Open.*

eMethods 2. Description of Adherence to the 32 Items of the Consolidated Criterion for Reporting Qualitative Research (COREQ)

Domain 1: Research Team and Reflexivity Statements

Qualitative interviews were conducted by the parent trial's research project manager who is trained in qualitative interviewing (TJS) and is also a female, registered nurse. Participants had frequently met the interviewer (TJS) prior to the interview as they were longitudinal participants in the parent trial. The interviewer had knowledge about the participants' trial and illness experience over the course of several years. One analyst was a female physician scientist with expertise in end-of-life decision-making (LJV); the second analyst was a female masters trained student of public health (EV). The lead researcher is the co-Director of the Qualitative and Mixed Methods Core at Penn State College of medicine and the second analyst (EV) is a research project manager who works full time for the qualitative and mixed methods core. Both are very experienced in qualitative research and mixed methods. At the outset of coding, analysts discussed intrinsic biases related to surrogate decision-making and bracketed these to the extent possible to maintain neutrality. Participants were informed of the goals of the research via IRB approved scripts prior to interview sessions.

Domain 2: Study Design

Interviews occurred either in the home environment, by phone, or at the medical center based on the participant's preference. No additional individuals were present during these interviews, which typically lasted 60 minutes, although the section for the interview was typically 5-20 minutes in duration. We took an ontological philosophical assumption that is appropriate for asking what is the nature of reality (in this case, the experiences around making a reported surrogate medical decision) as seen through the views of the spokespersons. We used a social constructivism framework that is appropriate when seeking understanding of an experience and its meaning.[1] In this study, we apply this framework to inductively generate a pattern of meaning related to the terms surrogate decision-maker and advocacy. We used a descriptive and inductive phenomenological approach to content analysis.[2,3] Additional details of our sample selection and analytic approach are provided in the manuscript body. The coding tree is provided in eTable1. The interview guide is provided in eMethod 2. Repeat interviews were not performed related to this analysis or research question. All interviews were audio recorded. The interviewer took field notes to help guide her reports of surrogate decisions to the research team, but these notes were not intended for incorporation into the analysis. Data saturation was judged to occur after review of three transcripts. Transcripts were not returned to participants due to study participation ending and feasibility issues.

Domain 3: analysis and findings

Coding details are found in the main manuscript. The coding tree is provided below (eTable1). The constant comparison method was for analysis and to derive emerging themes that were not identified in advance. Coding was performed using MAXQDA 2020. Major themes and quotations are presented in the manuscript. Minor themes and outlier results are also reported. Participants did nor check results due to study period ending.

---

[1] Creswell JW, Inquiry Q. *Research design: choosing among five approaches*. Sage, Thousand Oaks, California; 2007: p.24.
[2] Creswell JW, Creswell JD. *Research design: Qualitative, quantitative, and mixed methods approaches.* Sage publications; 2017.
[3] Moustakas C. *Phenomenological research methods.* Sage; 1994.

© 2022 Van Scoy LJ et al. *JAMA Network Open.*

eMethods 3. Visit 3 Interview Guide Revised Versions*

During this interview, I'm going to ask you some questions that will help us better understand the experience that you had since [INSERT EVENT (*e.g. John died; Betty was hospitalized; you made the decision to XYZ*)]. Specifically, I'm interested in hearing whether you had to make decisions, how that went, and how you felt during the decision-making process. I recognize that this may be difficult for you, so if you feel uncomfortable answering my questions or need to stop to take a break, please don't hesitate to let me know that.

1. During our phone interview, you told me that [*Patient*] recently [*INSERT EVENT; i.e. died, needed a decision made, etc.*] Tell me what happened. (PROMPT, if not answered already: What led up to the event?)

2. In thinking about that event, did you make a medical decision on behalf of your loved one? Tell me more about that.
   - When people are asked to make medical decisions for others, they are sometimes presented with a variety of choices and options. Were you presented with choices and options? If yes, tell me what the choices or options were. (Prompts: If they say "I had no choice"- probe further – "why did you feel like you had no choice?")
   - How did you ultimately arrive at a decision? (PROMPT if needed: What was going through your head as you made that decision? What (if anything) was helpful to you as you made decisions? What (if anything) was not helpful to you as you made decisions?
   - On what did you base your decisions? (If they do not understand the question: "If there were different voices in your head helping you to make the decision, what were they saying?")
   - Did anyone help you make decisions? If so, tell me more about that.

3. When it came time for you to make a decision on behalf of your loved one, how prepared did you feel? (PROMPT, if needed: Tell me more about that.
   - Prepared: If they felt prepared, would anything have helped you feel even *more* prepared? If so, what?
   - Not Prepared: If they did NOT feel prepared, what might have helped you feel better prepared? Explain.

   - Do you feel you understood what your loved one would have wanted regarding their medical treatment? (PROMPT, if needed: Tell me more about that.
   - How did you know what your loved one would have wanted?

4. Early on in this study, you or your loved one completed an advance directive on the computer.
   - In what way (if at all) did this process help you with your decision-making?
   - In what way (if at all) did this process help you to feel prepared to make decisions on behalf of your loved one?

5. People often say it's stressful to make medical decisions for other people. Thinking about the recent decision you made on behalf of your loved one, did you find that process to be stressful or distressing for you? Tell me more about that.

6. Do you feel that [patient's] end-of-life wishes were honored? Tell me more about that.

7. What (if anything) helped you feel prepared for [patient's] *death*? Do you feel that participating in this study helped you to feel prepared for [patient's death] in any way? If so, how?

8. What (if anything) were you not prepared for?

We're on to the last part of the interview. People sometimes have very different understandings of what it means to make a surrogate decision. I'd like to learn what this concept means to you.

© 2022 Van Scoy LJ et al. *JAMA Network Open.*

9. In your own words, please tell me what “making a surrogate medical decision” means to you.

10. Do you perceive that there is a difference between making a surrogate decision and being an advocate? If so, can you tell me more about that?

Thanks so much for sharing your story with me. Your input has been very valuable and we appreciate the opportunity to learn from your experiences. I just have one more question about your involvement in this study.

11. Thinking about the phone calls you received from us every few months, how (if at all) did these phone calls affect how you made decisions on behalf of your loved one?
12. What impact (if any) did these phone calls have on you as the designated surrogate decision maker for your loved one?

*This Interview Guide was written at fourth grade reading level but was not shared in print with participants. All questions were asked verbally.

© 2022 Van Scoy LJ et al. *JAMA Network Open.*

eTable. Coding Tree

Code	Definition
1. Specific Definitions of Surrogate Decision Making (SDM)	
1a. Facilitated decision making	Helping patient make decision when they don't know what to do
1b. Highest level of authority	SDM seen as being at the "top of the ladder" for decisions, hierarchical in nature
1c. Scientific in nature	SDM described as learning, reading, and deciding. No emotions involved, mechanical/scientific process
1d. Doing what is best for patient	Doing what is in the best interest of the patient
1e. Final decision maker	Being the person who makes the final decision, end of life decision maker, makes tough decisions
1f. Voice of patient	Knowing what is best and being patient's voice
1g. Fulfilling or respecting wishes	Knowing what the patient wants and doing it
1h. Other	Other definitions for SDM
2. Specific Definitions of Advocacy	
2a. Asking questions	Asking questions of medical team, gathering information, talking to doctor, reporting on patient health, etc.
2b Providing support	Providing emotional support, passionate involvement, being involved
2c. Advocating for life	Advocating to keep someone alive, described as directional in nature, fighting to keep someone alive
2d. Makes sure basic needs are met	Making sure patient has what they need (food, medication, etc) basic needs are met, includes tangible things
2e. Voice of patient	Being patient's voice, speaking up and telling the truth when the patient won't
2f. Decision maker on someone's behalf	Making tough decisions, making decisions on patient's behalf
2g. Fulfilling or respecting wishes	Explicit decision about patient's autonomy
2h. Doing what is best for patient	Fighting for the "right thing," lobbying for someone
2i. Other	Other definitions for advocacy
3. Advocacy and SDM are similar	
4. Other or Not sure where to code	

© 2022 Van Scoy LJ et al. *JAMA Network Open.*
