# Supplementary material for: Conceptualization of Surrogate Decision-making Among Spokespersons for Chronically Ill Patients
Source: JAMA Netw Open. 2022 Dec 8;5(12):e2245608. doi: 10.1001/jamanetworkopen.2022.45608 (PMC9856522; doi:10.1001/jamanetworkopen.2022.45608)
Supplement: Supplement 2. — Data Sharing Statement [file jamanetwopen-e2245608-s002.pdf]

Data Sharing Statement

Van Scoy. Conceptualization of Surrogate Decision-making Among Spokespersons for Chronically Ill Patients. *JAMA Netw Open*. Published December 08, 2022. doi:10.1001/jamanetworkopen.2022.45608

Data
Data available: Yes
Data types: Deidentified participant data
How to access data: Upon request from MPIs mgreen@pennstatehealth.psu.edu or blevi@pennstatehealth.psu.edu
When available: With publication

Supporting Documents
Document types: None

Additional Information
Who can access the data: researchers whose proposed use of the data has been approved
Types of analyses: for research purposs
Mechanisms of data availability: with MPI support and signed data access agreement.
